# Combination of anti-PD-1 antibody with P-GEMOX as a potentially effective immunochemotherapy for advanced natural killer/T cell lymphoma

**DOI:** 10.1038/s41392-020-00331-3

**Published:** 2020-12-30

**Authors:** Jun Cai, Panpan Liu, Huiqiang Huang, Yajun Li, Shuyun Ma, Hui Zhou, Xiaopeng Tian, Yuchen Zhang, Yan Gao, Yi Xia, Xuanye Zhang, Hang Yang, Lirong Li, Qingqing Cai

**Affiliations:** 1 State Key Laboratory of Oncology in South China, Collaborative Innovation Center of Cancer Medicine, Sun Yat-sen University Cancer Center, Guangzhou, 510060 P.R. China; 2 Department of Medical Oncology, Sun Yat-sen University Cancer Center, Guangzhou, 510060 P.R. China; 3 Department of Lymphoma and Hematology, Hunan Cancer Hospital, Changsha, 410013 P.R. China; 4 Department of Lymphoma and Hematology, The Affiliated Cancer Hospital of Xiangya School of Medicine, Central South University, Changsha, 410013 P.R. China

**Keywords:** Drug development, Haematological cancer, Drug development, Predictive markers, Haematological cancer

## Abstract

Advanced natural killer/T cell lymphoma (NKTL) has demonstrated poor prognosis with currently available therapies. Here, we report the efficacy of anti-programmed death 1 (PD-1) antibody with the P-GEMOX (pegaspargase, gemcitabine, and oxaliplatin) regimen in advanced NKTL. Nine patients underwent six 21-day cycles of anti-PD-1 antibody (day 1), pegaspargase 2000 U/m^2^ (day 1), gemcitabine 1 g/m^2^ (days 1 and 8) and oxaliplatin 130 mg/m^2^ (day 1), followed by anti-PD-1 antibody maintenance every 3 weeks. Programmed death-ligand 1 (PD-L1) expression and genetic alterations were determined in paraffin-embedded pretreatment tissue samples using immunohistochemistry and next-generation sequencing (NGS) analysis. Responses were assessed using ^18^F-fluorodeoxyglucose positron emission tomography (^18^FDG-PET) and computed tomography or magnetic resonance imaging. Eight patients exhibited significant responses, comprising of seven complete remissions and one partial remission (overall response rate: 88.9%). After a median follow-up of 10.6 months, 6/9 patients (66.7%) remained in complete remission. The most common grade 3/4 adverse events were anemia (33.3%), neutropenia (33.3%), and thrombocytopenia (33.3%); all of which were manageable and resolved. Immunochemotherapy produced a high response rate in patients with positive PD-L1 expression (5/6, 83.3%). NGS analysis suggested that *STAT3/JAK3/PD-L1* alterations and *ARID1A* mutation were associated with immunochemotherapy efficacy. Mutation in *DDX3X* and alteration in epigenetic modifiers of *KMT2D*, *TET2*, and *BCORL1* might indicate a poor response to immunochemotherapy. In conclusion, the anti-PD-1 antibody plus P-GEMOX regimen demonstrated promising efficacy in advanced NKTL. PD-L1 expression combined with specific genetic alterations could be used as potential biomarkers to predict therapeutic responses to immunochemotherapy.

## Introduction

Natural killer/T-cell lymphoma (NKTL) is a well-characterized subtype of peripheral T-cell lymphoma that is more common in East Asia and Latin America.^1,2^ More than two-thirds of NKTL patients have stage I or II diseases in the upper aerodigestive tract at the time of diagnosis.^3,4^ The prognosis of this subgroup of patients has been significantly improved with the use of concurrent chemoradiotherapy or sequential chemoradiotherapy with non-anthracycline chemotherapy.^5–7^ In contrast to localized NKTL where front-line therapy may be associated with long-term remission in over 60% of patients, the optimal treatment for advanced NKTL remains a major challenge as 70–80% of the patients experience disease progression or death within 5 years of diagnosis.^8–11^

Asparaginase and pegaspargase are key components of chemotherapeutic regimens for advanced NKTL.^12–14^ However, treatment-related adverse events (AEs) still remain a significant challenge. Several studies have suggested that pegaspargase, gemcitabine, and oxaliplatin (P-GEMOX) might have high efficacy while exhibiting better tolerability, and is recommended as the first-line treatment. In a retrospective study of 10 years’ real-world clinical experience in the treatment of NKTL from China, the P-GEMOX regimen provided an overall response rate (ORR) of 71.7% in advanced NKTL, with a 2-year progression-free survival (PFS) rate of 33.8%, and a 2-year overall survival (OS) rate of 44.5%.^15^ In addition, a recent prospective study by Huang et al.^16^ showed that P-GEMOX plus thalidomide regimen had an ORR of 87.1% and a complete response (CR) rate of 56.3% in advanced or relapsed/refractory (r/r) NKTL, with a 3-year PFS and OS of 47.0% and 44.3%, respectively. However, it is also important to note that ~70% of patients would still relapse despite first-line chemotherapy. Currently, the long-term survival rate of patients with advanced NKTL is still low. Thus, new drugs and effective therapeutic approaches are urgently needed.

NKTL has a high frequency of programmed death-ligand 1 (PD-L1) expression, which is upregulated by the Epstein–Barr virus (EBV),^17,18^ making NKTL a target for anti-programmed death 1 (anti-PD-1)/PD-L1 antibodies.^19,20^ Several studies have reported that the single-agent anti-PD-1 antibody could provide an ORR of 57.1–100% in r/r NKTL, with a 1-year OS rate of 82.1%.^21–25^ Further, those encouraging results on anti-PD-1 antibody in NKTL have started to challenge the current treatment paradigms of NKTL and have provided the rationale for evaluating PD-1 blockade as a first-line therapy of patients with advanced NKTL.

More recently, as first-line therapy, anti-PD-1 antibody combined with chemotherapy has shown benefits in solid tumors. In the KEYNOTE-407 trial, treatment with pembrolizumab plus chemotherapy was found to be superior than chemotherapy alone for squamous non-small-cell lung cancer in terms of PFS and OS.^26^ In the KEYNOTE-048 study, pembrolizumab plus chemotherapy demonstrated superior OS compared with cetuximab plus chemotherapy for recurrent or metastatic head and neck squamous cell carcinoma.^27^

Considering the potential synergistic efficacy of immune checkpoint inhibitor and chemotherapy, we propose a novel treatment strategy by combining anti-PD-1 antibody with P-GEMOX for advanced NKTL and herein report its treatment efficacy, safety, and exploratory biomarker results.

## Results

### Patients

**Fig. 1:**
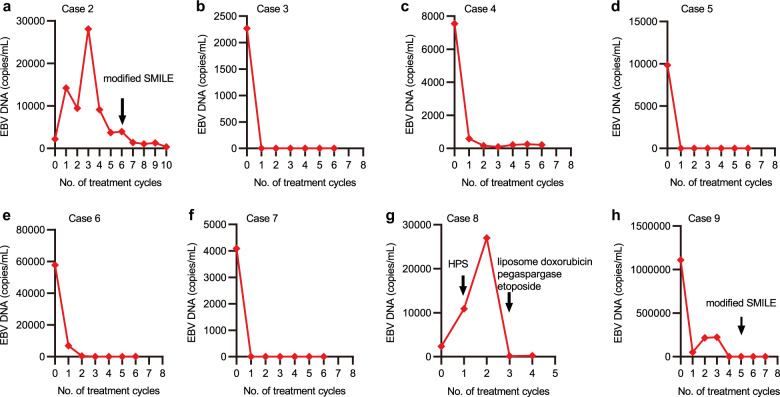
Changes in circulating EBV DNA during immunochemotherapy. **a** The EBV DNA in case 2 increased from 2220 to 28,100 copies/mL after 3 cycles of immunochemotherapy, and then became normal after salvage therapy of modified SMILE. **b**–**f** The EBV DNA in cases 3, 4, 5, 6, and 7 became normal after 1 or 2 cycles of immunochemotherapy. **g** The EBV DNA in case 8 increased from 2350 to 27,000 copies/mL after immunochemotherapy, and became normal after salvage therapy of etoposide, pegaspargase, and liposome doxorubicin. **h** The EBV DNA in case 9 decreased after the first cycle of immunochemotherapy, and then increased. EBV DNA then fell to 0 copies/mL after salvage therapy of modified SMILE

A total of nine patients diagnosed with advanced NKTL were treated and included in this study. Their median age was 38 (range, 22–65) years. Circulating EBV DNA ranged from 2220 to 1,110,000 copies/mL (Fig. 1) and Epstein–Barr virus-encoded RNA (EBER) was pathologically confirmed as positive in all cases. The patients’ characteristics are summarized in Table 1.

**Table 1:** Clinical characteristics of nine patients with NKTL treated with the immunochemotherapy regimen

Case no.	Age, y	Sex	Stage	Primary site	BM	HPS	ECOG PS	PINK/PINK-E score	Extra-nodal sites	B symptoms	LDH/ULN	HBsAg	Baseline HBV DNA level (IU/mL)
1	43	F	IV	Nasopharynx, lymph nodes	–	Nil	1	2/3	1	Nil	0.76	+	119
2	22	F	IV	Nasopharynx, tonsils	+	Nil	1	1/2	2	Yes	1.01	–	0
3	35	M	IV	Nasal cavities, sinuses, sternal manubrium	–	Nil	1	1/2	2	Nil	0.55	+	238
4	28	M	IV	Nasal cavity, subcutaneous nodules, lymph nodes	–	Nil	1	2/3	>3	Yes	1.30	–	0
5	62	M	IV	Nasopharynx, nasal cavity, tonsils, lymph nodes, small intestine	–	Nil	1	2/3	1	Nil	0.96	–	0
6	57	F	IV	Subcutaneous nodules, lymph nodes	–	Nil	1	3/4	>5	Yes	0.65	–	0
7	65	M	IV	Nasal cavities, sinus, liver, spleen, lymph nodes, bones	–	Nil	1	3/4	>3	Yes	1.4	–	0
8	38	M	IV	Nasal cavities, sinuses, tonsil, lymph nodes	–	Yes	1	1/2	1	Yes	1.8	+	942
9	29	F	IV	Nasopharynx, lymph nodes, bones	–	Yes	1	2/3	7	Yes	8.8	–	0

*F* female, *M* male, *BM* Bone marrow, *HPS* hemophagocytic syndrome, *ECOG* *PS* Eastern Cooperative Oncology Group Performance Status, *PINK/PINK-E* prognostic index of natural killer lymphoma/prognostic index of natural killer lymphoma–Epstein–Barr virus, *LDH* lactate dehydrogenase isoenzyme (120–250 U/L), *ULN* upper limit of normal, *HBsAg* hepatitis B surface antigen, *HBV DNA* hepatitis B virus-deoxyribonucleic acid

### Response to the anti-PD-1 antibody plus P-GEMOX regimen

After a median of 5 (range, 1–7) cycles of the prescribed proposed immunochemotherapy regimen, objective response was observed in eight patients. The ORR for the nine treated patients was 88.9%, including 7 CRs (77.8%) and 1 partial response (PR, 11.1%) (Table 2 and Supplementary Fig. 1). The 1-year PFS rate was 66.7% and the 1-year OS rate was 100% (Supplementary Fig. 2).

**Table 2:** Therapies and outcomes of nine patients with NKTL treated with the immunochemotherapy regimen

Case no.	TPS	CPS	Treatment regimens (cycles)	Anti-PD-1 antibody dose, mg	Best overall response	PFS, months
1	NA	NA	P-GEMOX (4), P-GEMOX+Chidamide (2), P-GEMOX+Pembrolizumab (2), Sintilimab (8)	200	CR	15.5
2	8	10	P-GEMOX (1); P-GEMOX+Toripalimab (4)	240	CR	3.8
3	30	45	P-GEMOX (1); P-GEMOX+Sintilimab (5); sequential RT with concurrent Sintilimab (2); Sintilimab (5)	200	CR	14.0
4	40	45	P-GEMOX+Sintilimab (3); P-GEMOX+Camrelizumab (3)	200 200	CR	11.8
5	98	100	P-GEMOX (1); P-GEMOX+Sintilimab (1); P-GEMOX (2) with sequential RT; P-GEMOX (3)	200	CR	10.6
6	10	13	P-GEMOX+Sintilimab (6); Sintilimab (1)	200	CR	9.4
7	NA	NA	P-GEMOX+Sintilimab (6)	200	CR	7.5
8	50	55	P-GEMOX+Sintilimab (1)	200	PD	0.8
9	0	0	P-GEMOX (2); P-GEMOX+Sintilimab (2)	200	PR	2.9

*TPS* tumor proportion score, the number of PD-L1 positive tumor cells (showing partial or complete membrane PD-L1 staining at any intensity) divided by the total number of tumor cells ×100; *CPS* combined positive score, the number of PD-L1 positive cells (tumor cells, lymphocytes, macrophages) divided by the total number of tumor cells ×100, *NA* not available, *P-GEMOX* pegaspargase, gemcitabine and oxaliplatin, *RT* radiotherapy, *CR* complete remission, *PR* partial remission, *PD* progression of disease, *PFS* progress-free survival

**Fig. 2:**
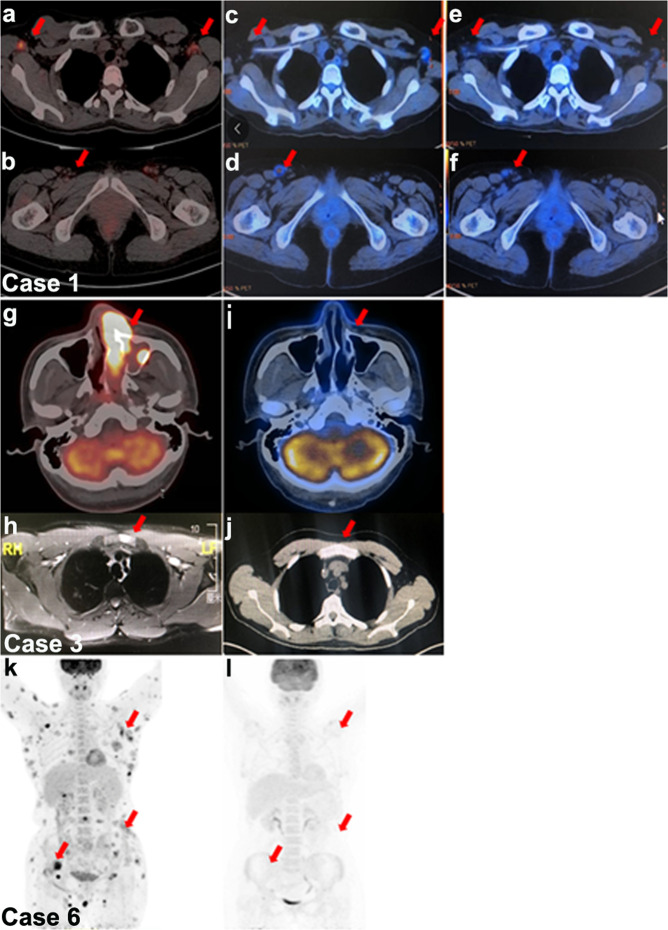
Imaging results of three representative patients. **a**–**f** PET/CT results of case 1. Baseline PET/CT showed multiple lymph nodes involvement (**a**, **b**). After the sixth cycle of conventional chemotherapy, there were persistent FDG-avid lesions by PET/CT scan (**c**, **d**). After 2 cycles of immunochemotherapy, no FDG-avid lesions were observed and CR was confirmed (**e**, **f**). **g**–**j** PET/CT and MRI results of case 3. Baseline PET/CT and MRI examination showed neoplastic metabolic lesions invading the nasal cavities and sinuses, with MRI-proved lymphomatous infiltration in the sternal manubrium (**g**, **h**). After 4 cycles of immunochemotherapy, no FDG-avid lesions were found in a follow-up PET/CT scan, and the manubrium sternum lesions completely disappeared; demonstrating CR (**i**, **j**). **k**, **l** PET/CT results of case 6. Baseline PET/CT scan of case 6, presenting no nasal disease but systemic tumor invasion of the skin and lymph nodes (**k**). After 6 cycles of immunochemotherapy, all original skin lesions resolved, and no FDG-avid lesions were seen in the PET/CT scan, which indicated a response of CR (**l**)

We first explored the potential impact of the immunochemotherapy regimen in a woman presented with nasopharyngeal and systemic lymph node involvement. She showed no response to P-GEMOX and P-GEMOX plus chidamide therapy. Then, anti-PD-1 antibody was given as a combination with the P-GEMOX regimen. All the lesions responded well, and a CR was observed at the end of the second course of the immunochemotherapy regimen (Fig. 2a–f). We next evaluated the immunochemotherapy regimen in another eight advanced patients, of whom seven demonstrated promising responses (CR, six patients; PR, one patient). Images of two patients (cases 3 and 6) with objective responses are shown in Fig. 2g–l.

After a median follow-up of 10.6 (range, 7.5–21.9) months, six patients remained in CR, and three experienced disease progression after achieving their best response. Case 2 achieved a CR after 4 cycles of immunochemotherapy, but before the fourth cycle, there was an increase in EBV DNA (Fig. 1a) and then bone marrow aspiration showed tumor cells reappearance in the bone marrow. The PFS of case 2 was 3.8 months. Case 9 presented with involvement of the nasopharynx, systemic bones, and lymph nodes of the neck and axilla. She developed hemophagocytic syndrome (HPS) during the first cycle of treatment. And then, her EBV DNA decreased (Fig. 1h), HPS was resolved and positron emission tomography-computed tomography (PET/CT) confirmed a PR after 2 cycles of immunochemotherapy. However, after 4 cycles of immunochemotherapy, her EBV DNA was found to be increased and PET/CT provided confirmatory evidence of disease progression. The PFS for case 9 was 2.9 months. Case 8 showed recurrent fever and facial midline destructive lesions, with PET/CT demonstrating involvement of the nasal cavities, sinuses, tonsils, and cervical lymph nodes. After the first cycle of immunochemotherapy, the EBV DNA significantly increased (Fig. 1g), HPS occurred, and the facial lesions progressed. The patient was then prescribed with the etoposide, pegaspargase, and liposome doxorubicin treatment. After 1 cycle, the HPS resolved, the facial lesions improved, and EBV DNA became undetectable (Fig. 1g). During anti-PD-1 antibody maintenance, two patients experienced pseudoprogression. The related details are shown in Supplementary Materials.

### Adverse events

All nine patients experienced treatment-related AEs. The most common grade 3 or 4 AEs were anemia (3 [33.3%] patients), neutropenia (3 [33.3%] patients), and thrombocytopenia (3 [33.3%] patients). Only one patient (case 4) developed immune-mediated AE of grade 2 hypothyroidism (Supplementary Table 1). No patients discontinued the treatment because of treatment-related AEs and all the AEs were manageable and resolved. Three patients with hepatitis B virus (HBV) infection received oral antiviral treatment (entecavir) and regular monitoring of HBV DNA. No patients had HBV outbreak or reactivation during the treatment. At data cutoff, their HBV DNA load all dropped to 0 IU/mL with the continuous and effective antiviral therapy.

### Expression of PD-L1

**Fig. 3:**
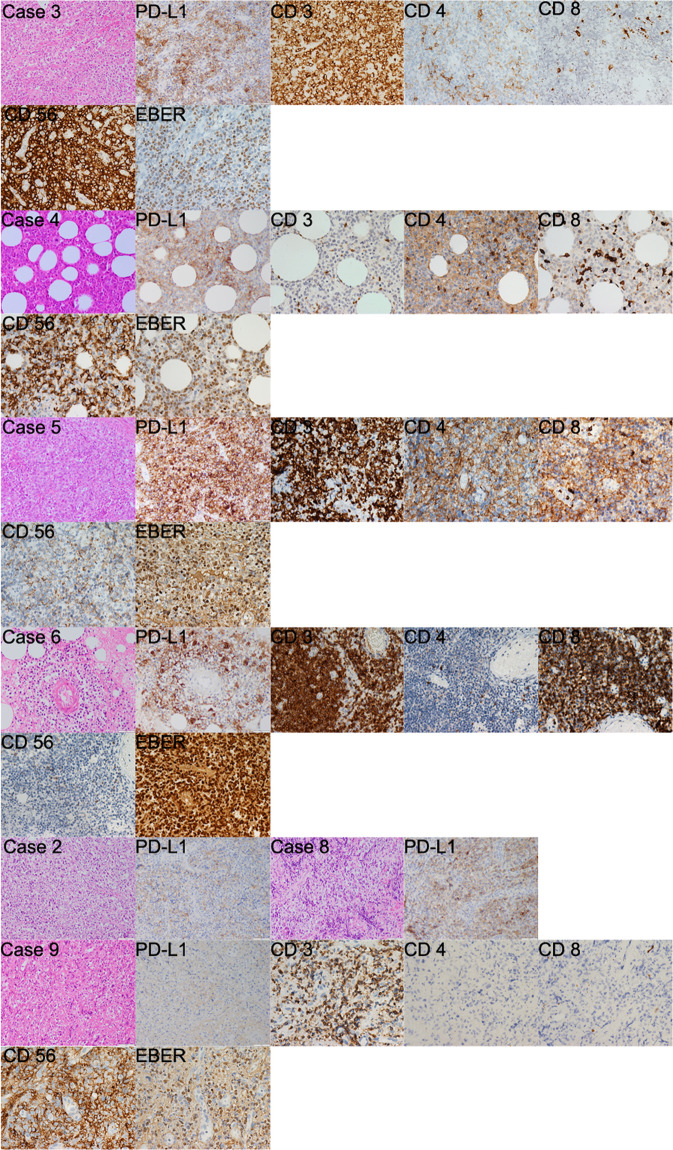
Immunohistochemistry (IHC) and hematoxylin/eosin (HE) staining of tumor specimens from seven NKTL patients. The expression of PD-L1, CD3, CD4, CD8, CD56, and Epstein–Barr virus-encoded RNA (EBER) in the clinical samples was analyzed by IHC staining (original magnification ×400). Due to limited tissue slides available for immunostaining, some of the specimens were only stained for the selected markers as indicated. PD-L1 was negative for case 9. EBER was positive in all seven cases. The expression of PD-L1 was only stained for cases 2 and 8

Immunohistochemistry (IHC) examination of PD-L1 expression and pathological markers are shown in Fig. 3 and Supplementary Table 2. Six (85.7%) patients (cases 2, 3, 4, 5, 6, and 8) demonstrated positivity of PD-L1 expression (Table 2). Five of them responded to immunochemotherapy and attained CR. Only one patient (case 8) with a high expression of PD-L1 was insensitive to immunochemotherapy. An absence (case 9) of tumor PD-L1 expression was found correlated with inferior response. Data were not available for PD-L1 IHC analysis in two patients (cases 1 and 7).

### Targeted next-generation sequencing-based mutation profiling of tissues

**Fig. 4:**
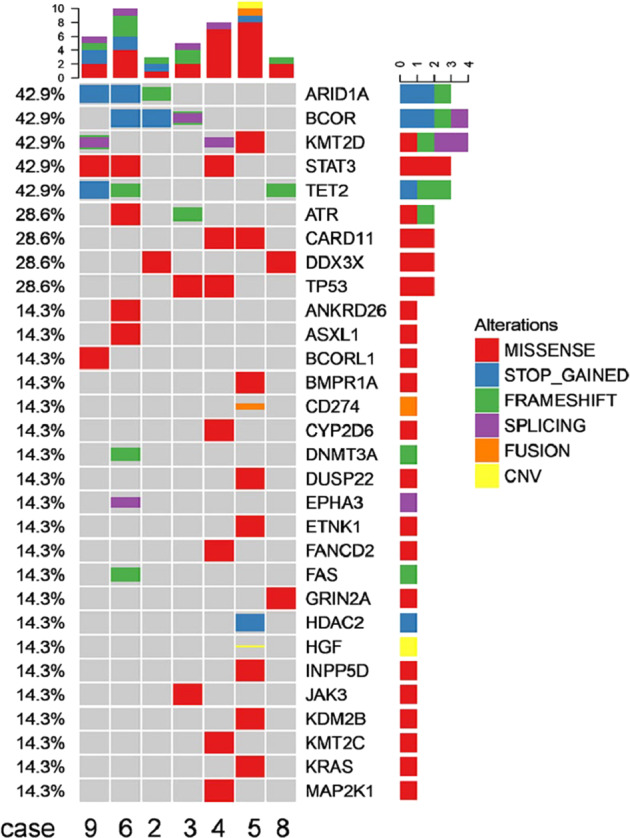
Heatmap for altered genes in the seven NKTL patients

As mentioned above, not all patients with positive PD-L1 expression responded to the immunochemotherapy regimen. To further explore the molecular mechanism of immunochemotherapy in advanced NKTL, 446 lymphoma- and cancer-relevant genes using next-generation sequencing (NGS) were analyzed in seven patients with available tumor tissues. Figure 4 shows the heatmap of their gene mutation profiles. The majority of gene alterations were missense mutations and a dominant G:C to A:T transition (Supplementary Fig. 3). The top five most frequently mutated genes were *STAT3* (cases 4, 6, 9; 42.9%, 3/7), *KMT2D* (cases 4, 5, 9; 42.9%, 3/7), *ARID1A* (cases 2, 6, 9; 42.9%, 3/7), *BCOR* (cases 2, 3, 6; 42.9%, 3/7), and *TET2* (cases 6, 8, 9; 42.9%, 3/7). Mutations for *DDX3X* was 28.6% (cases 2, 8; 2/7), *TP 53* was 28.6% (cases 3, 4; 2/7), and *CD274/PD-L1* was 14.3% (case 5, 1/7). Among the above changes, the *STAT3* activating mutation in cases 4 and 6 was associated with PD-L1 overexpression and good response, whereas no PD-L1 expression was detected in case 9 who exhibited poor response. *ARID1A* mutations (STOP-gained and frame-shift) were observed in cases 2, 6, and 9. The two patients (cases 2 and 8) who had *DDX3X* mutations experienced disease progression. The third patient with poor clinical outcome (case 9) exhibited mutations mainly at the epigenetic modifiers, as previously defined.^28^ Gene Ontology–Biological Process (GO–BP) enrichment analysis revealed that the T-cell receptor signaling pathway, immune response-regulating pathway, and immune response-activating pathway were enriched in the group without disease progression (cases 3, 4, 5, and 6) (Supplementary Fig. 4).

## Discussion

This is the first time that the role of immune checkpoint inhibition plus pegaspargase-based chemotherapy in advanced NKTL has been evaluated. Our results suggested that the addition of an anti-PD-1 antibody to the P-GEMOX regimen could be a highly effective combination for patients with advanced NKTL and possessed a favorable safety profile. The dynamic change in circulating EBV DNA was associated with therapeutic efficacy, which proved to be a significant predictor of response.

In addition to being a safe and effective regimen for NKTL treatment, P-GEMOX may also have an immune-enhancing effect. Gemcitabine, a nucleoside analog included in the P-GEMOX regimen, has been found to reduce the amount of circulating myeloid-derived suppressor cells (MDSCs), favoring the reprogramming of tumor-associated macrophages toward an immunostimulatory phenotype.^29–31^ Besides such direct immunostimulatory effects, gemcitabine could also stimulate the expression of major histocompatibility complex (MHC) class I molecules in cancer cells, thereby increasing their antigenicity.^32^ Oxaliplatin (a platinum derivative also included in the P-GEMOX regimen) has been shown to be able to promote immunogenic cell death by increasing the CD8^+^ cytotoxic T lymphocytes (CTLs)/CD4^+^CD25^+^FOXP3^+^ regulatory T (T_REG_) cell ratio and depleting MDSCs, as well as promoting the activity of neutrophils and macrophages. Thereby, oxaliplatin treatment can generate a robust effector immune response.^33,34^ Combining immunogenic P-GEMOX chemotherapy with PD-1 blockade theoretically drives deeper and sustained tumor responses. In this present study, 4 types of anti-PD-1 antibodies were used. Although bonded to different target epitopes at the PD-1 molecule,^35–38^ they had the same mechanism of action (inhibiting the PD-1/PD-L1 immune checkpoint, leading to restoration of T-cell functions against cancer cells), and thus would not significantly affect the interpretation of the study results. This notion is supported by our observation that eight out of the nine investigated patients treated with different anti-PD-1 antibodies plus P-GEMOX showed good clinical responses, including 7 CRs and 1 PR.

The major advantage of this regimen is that it does not require hematopoietic stem cell transplantation (HSCT) as a post-remission consolidation therapy. The role of HSCT in NKTL has been controversial. Evidence supporting the use of HSCT (autologous-HSCT or allogenic-HSCT) is mainly based on the results of uncontrolled retrospective studies, and there are no randomized studies that directly compared post-remission consolidation HSCT versus observation for NKTL. Survival benefits from HSCT were suggested by a retrospective analysis that compared 47 patients who underwent autologous-HSCT with 107 historical controls.^39^ For patients who attained CR, the 5-year disease-specific survival of HSCT group was higher than controls (87.3% versus 67.8%). However, conclusions should be interpreted with caution because the study did not match the groups for the chemotherapeutic regimen or timing of radiotherapy. Another study that included 21 patients with advanced disease reported no apparent difference in long-term outcomes between autologous-HSCT group versus historical controls without post-remission treatment.^40,41^ Given the high toxicity and uncertainty of efficacy in NKTL consolidation, HSCT was not performed in our study. Besides, prior data showed that the anti-PD-1 antibody exhibited durable antitumor activity in patients receiving this therapy, which persisted after drug discontinuation. Thus, the patients in our study received prolonged anti-PD-1 antibody maintenance aiming to induce long-term remission and survival.

Our study had a CR rate of 77.8%. In previously reported studies on asparaginase or pegaspargase-containing regimens, such as the SMILE and AspaMetDex studies,^12,16^ the observed CR rates in advanced NKTL were 40.0% and 38.5%, respectively. The CR rate of our immunochemotherapy seems to be better than that of SMILE and AspaMetDex, however, future randomized controlled trials are still needed to verify this conclusion. In a recent randomized controlled multicenter study reported by Huang et al.,^16^ 31 patients with stage III/IV or r/r NKTL achieved an ORR of 87.1% and a CR rate of 56.3% after P-GEMOX plus thalidomide therapy. Attaining a CR has been found to be a significant factor impacting on the outcome of NKTL. In our present study, 77.8% of patients achieved CR, suggesting a better outcome could be observed during future long-term follow-up.

The immunochemotherapy regimen demonstrated an acceptable safety profile in patients with advanced NKTL. The most common AEs in our study were emesis (44.4%), elevated transaminase (44.4%), nausea (33.3%), prolonged activated partial thromboplastin time (APTT, 33.3%), grade 3 or 4 AEs including anemia (33.3%), neutropenia (33.3%), and thrombocytopenia (33.3%). The hematologic toxicities seen in our study were slightly higher than that reported with the P-GEMOX regimen, which rendered 24.5% of grade 3 or 4 neutropenia, 10.6% of grade 3 or 4 anemia, and 15.8% of grade 3 or 4 thrombocytopenia.^16^ Great importance should be attached to hematologic toxicities related to this regimen. Neither pneumonia nor allergy was observed. No increase in hepatocyte transaminase due to virus replication or reactivation occurred in the three patients with HBV infection. In the present study, no discontinuation or death caused by treatment-related AEs was observed and all the AEs were manageable and reversible.

Apart from the exploration of the most efficient combination of therapy, identification of biomarkers to select patients who could benefit from PD-1 inhibition is also required. PD-L1 expression results were evaluable in seven patients, of whom six were found positive. PD-L1 positive patients had a higher response rate (CR, *n* = 5). Case 9, who was refractory to the immunochemotherapy regimen, showed no detectable PD-L1 expression. Dynamic monitoring of circulating EBV DNA value suggested that EBV DNA copy number was associated with the tumor activity and predictive of the treatment efficacy. During the course of the immunochemotherapy regimen, a significant reduction in plasma EBV DNA was observed in cases 3, 4, 5, 6, and 7. A rapid increase in plasma EBV DNA levels was associated with disease progression, while a decrease was associated with the control of the disease; which was observed in cases 2, 8, and 9.

This study is the first to have explored genes alteration in advanced NKTL and their potential roles as biomarkers in response to PD-1 inhibitor combined chemotherapy treatment. Our NGS results suggested that the *STAT3/JAK3/PD-L1* alterations were associated with immunochemotherapy. This is consistent with the observation that *STAT3* mutation could increase the phosphorylation of STAT3, and could thus enhance the transcription activity of STAT3, leading to elevated PD-L1 expression by binding to the promoter of PD-L1 gene.^42^ Recently, a study^28^ stratified NKTL into 3 molecular subtypes using multi-omics analysis. In this present study, case 9 had negative PD-L1 expression (IHC score, 0). Mutations in *ARID1A*, *KMT2D*, *TET2*, and *BCORL1* for case 9 suggested deregulation of the epigenetic control of transcription. This patient seemed to belong to the HEA subtype^28^ and was more sensitive to the histone deacetylase inhibitors. *ARID1A* is a tumor suppressor gene and several studies showed that *ARID1A* mutations were associated with increased immune activity and could predict benefits from checkpoint blockade in solid tumors.^43–45^ In our study, case 9 was negative for PD-L1 expression but also responded to the immunochemotherapy, suggesting that such positive response might be attributed to *ARID1A* alteration. Case 8 had higher expression of PD-L1 but was resistant to PD-1 inhibition. This negative result could be due to *DDX3X* mutation since it was previously shown that *DDX3X* mutation was associated with poor prognosis.^28,46^ Further, a recent study showed that *DDX3X* mutation could affect cell-fate decisions in cells under stress conditions by regulating NLRP3 inflammasome.^47^ In our study, the *DDX3X* mutation might change the response of NKTL to the stress induced by anti-PD-1 antibody and P-GEMOX, leading to the blocking of lymphoma cell death and drug resistance. Overall, our findings suggested that mutations in *STAT3*, *ARID1A*, and *DDX3X* might be important molecular events that could be combined with PD-L1 expression to predict clinical response to immunochemotherapy.

There were several limitations worth mentioning. First, the investigated cohort comprised of a relatively small sample size. Second, the biomarker analysis of PD-L1 expression and tumor mutations could have been insufficiently powered due to the limited biopsy samples available. Third, the antitumor activity analyses were preliminary. Thus, a larger sample size prospective study with longer follow-up time is still required for biomarker, PFS, and OS assessment to validate the study’s findings.

Based on the results of this exploratory study, we initiated a multicenter, single-arm, phase II trial (NCT04127227) consisting of sintilimab combined with P-GEMOX for untreated advanced NKTL patients. Future results of this prospective study could shed more light on the efficacy and safety profile of immunochemotherapy in this patients’ category. Molecular biology and genetic analysis could further help to differentiate patients based on their predicted response to immunochemotherapy.

## Materials and methods

### Patients and treatment

From July 6, 2018, to September 11, 2019, a total of nine patients with advanced NKTL received anti-PD-1 antibody with P-GEMOX at the Sun Yat-sen University Cancer Center (Guangzhou, China) and Hunan Cancer Hospital (Changsha, China). Patients with advanced NKTL received six cycles of anti-PD-1 antibody (day 1), pegaspargase 2000 U/m^2^ (day 1), gemcitabine 1 g/m^2^ (days 1 and 8), and oxaliplatin 130 mg/m^2^ (day 1) every 3 weeks. Those who achieved an objective response received anti-PD-1 antibody maintenance once every 3 weeks (Supplementary Fig. 5). Eligible patients were aged 18 years and older, and had adequate organ and bone marrow function. Patients with central nervous system involvement and secondary tumors were excluded. Patients with HBV infection were required to be receiving effective and continuous antiviral therapy (entecavir), and have a viral load of <1000 IU/mL at baseline.

### Response assessment

Scheduled ^18^F-fluorodeoxyglucose (^18^FDG) PET/CT or magnetic resonance imaging (MRI) was performed. Plasma samples were collected every 3 weeks to dynamically monitor circulating EBV and HBV load by quantitative polymerase chain reaction (PCR). Response assessment was made every 6 weeks according to standard criteria (5-point Deauville score).^48^ During anti-PD-1 antibody maintenance therapy, response assessment was made every 3 months using the Lymphoma Response to Immunomodulatory Therapy Criteria (LYRIC).^49^ The National Cancer Institute Common Terminology Criteria for Adverse Events, version 5.0 was used to grade AEs.

### Immunohistochemistry analysis

Paraffin-embedded pretreatment tissue samples were obtained from seven patients. Expression of tumoral PD-L1 was determined by IHC using mouse monoclonal antibody clone 22c3. Combined positive score (CPS) was defined as the number of PD-L1 positive cells (tumor cells, lymphocytes, macrophages) divided by the total number of tumor cells ×100; Tumor proportion score (TPS) was defined as the number of PD-L1 positive tumor cells (showing partial or complete membrane PD-L1 staining at any intensity) divided by the total number of tumor cells ×100. An expression percentage of 5% was applied as the threshold cutoff for PD-L1 positivity.^50^

### Next-generation sequencing analysis

Genomic DNA from formalin-fixed paraffin-embedded tissue sections was extracted with QIAamp DNA FFPE Tissue kit (Qiagen). Sequencing libraries were prepared using the KAPA Hyper Prep Kit (KAPA Biosystems) according to manufacturer’s instructions for different sample types. Customized xGen lockdown probes (Integrated DNA Technologies) targeting 446 leukemia- and lymphoma-related genes were used for hybridization enrichment. Capture reaction was performed with Dynabeads M-270 (Life Technologies) and xGen Lockdown hybridization and wash kit (Integrated DNA Technologies) according to manufacturers’ protocols. Genomic DNA was extracted for NGS analysis. All samples subjected to NGS analysis were required to have >10% of tumor cells as identified by immunohistochemistry.

### Sequencing data analyses

Mutation Calling Trimmomatic was used for FASTQ file quality control. Leading/trailing low quality (quality reading below 20) or N bases were removed. Paired-end reads were then aligned to the reference human genome (build hg19) using the Burrows–Wheeler Aligner (BWA). PCR deduplication was performed using Picard and local realignment around indels and base quality score recalibration were performed using GATK3. Unfortunately, matched germline DNA of patients as a normal control for mutation analysis was not available for this retrospective study. Somatic mutations were first called for each sample (the filtering criteria were a variant frequency of ≥0.5% and 5 or more supporting reads from both directions). Common single-nucleotide polymorphisms were excluded if they were present in >1% of the population frequency in the 1000 Genomes Project or the Exome Aggregation Consortium 65,000-exome database. The resulting mutation list was further filtered by an in-house list of recurrent artifacts and common single-nucleotide polymorphisms based on ~500 whole blood samples (normal pool) from Chinese patients with cancer that were sequenced with the same gene panel at an average depth of 400×. All SNVs/indels were annotated with ANNOVAR, and each SNV/indel was manually checked on the Integrative Genomics Viewer (IGV). Copy number variations (CNVs) were detected using in-house-developed software.

### Ethical consideration

All patients were informed of possible AEs and provided informed consent for the treatment. The study was approved by the Institutional Review Board of Sun Yat-sen University Cancer Center (No. B2020-163-01).

## Supplementary information

Supplementary Materials

## Data Availability

The authenticity of this article has been validated by uploading the key raw data onto the Research Data Deposit public platform (www.researchdata.org.cn), with the approval RDD number as RDDA2020001527.
