# Supplementary material for: Combination of anti-PD-1 antibody with P-GEMOX as a potentially effective immunochemotherapy for advanced natural killer/T cell lymphoma
Source: Signal Transduct Target Ther. 2020 Dec 30;5:289. doi: 10.1038/s41392-020-00331-3 (PMC7772337; doi:10.1038/s41392-020-00331-3)
Supplement: Supplementary file 1 — Supplementary Materials [file 41392_2020_331_MOESM1_ESM.docx]

Supplementary Materials for

Combination of anti-PD-1 antibody with P-GEMOX as a potentially effective immunochemotherapy for advanced natural killer/T cell lymphoma

Jun Cai^1,2^, Panpan Liu^1,2^, Huiqiang Huang^1,2^, Yajun Li^3,4^, Shuyun Ma^1,2^, Hui Zhou^3,4^, Xiaopeng Tian^1,2^, Yuchen Zhang^1,2^, Yan Gao^1,2^, Xuanye Zhang^1,2^, Hang Yang^1,2^, Lirong Li^1,2^, Qingqing Cai^1,2*^

Correspondence to: caiqq@sysucc.org.cn

**This PDF file includes:**

Materials and Methods

Supplementary Text

Figures. S1 to S5

Tables. S1 to S2

Materials and Methods

Anti-PD-1 antibodies used in the study

Pembrolizumab, administered intravenously at a dose of 200 mg every 3 weeks;

Manufacturer: Merck & Co., Inc. Kenilworth, New Jersey, U.S.A.

Sintilimab, administered intravenously at a dose of 200 mg every 3 weeks;

Manufacturer: Innovent Biologics (Suzhou, China) Co., Ltd.

Toripalimab, administered intravenously at a dose of 240 mg every 3 weeks;

Manufacturer: Junshi Biosciences (Shanghai, China) Co., Ltd.

Camrelizumab, administered intravenously at a dose of 200 mg every 3 weeks;

Manufacturer: Jiangsu Hengrui Medicine (Lianyungang, China) Co., Ltd.

Supplementary Text

Pseudoprogression during anti-PD-1 antibody maintenance

In case 1 patient, after 2 cycles of anti-PD-1 antibody maintenance, there was an increase in the size and metabolic activity of preexisting lymph nodes lesions according to the PET/CT scan. Without any clinical deterioration, it was defined as pseudoprogression according to LYRIC,^1^ and anti-PD-1 antibody maintenance continued. case 6 was initially presented with non-nasal involvement but rapidly progressive cutaneous ulceration. The original skin lesions appeared again after two cycles of anti-PD-1 antibody maintenance. Defined as pseudoprogression, anti-PD-1 antibody maintenance continued and by the end of the data cutoff, she was still asymptomatic with all existed lesions remaining stable without progression.

Salvage treatment after failing anti-PD-1 antibody with P-GEMOX regimen

After failing anti-PD-1 antibody with P-GEMOX regimen, Case 2 patient received modified SMILE (ifosfamide 5g/m2 [cumulative dose], etoposide 300mg/m2 [cumulative dose], dexamethasonea 15mg/m2 [day 1-5], pegaspargase 2000 U/m2 [day 7], methotrexate 3g/m2 [day 5]) regimen as salvage therapy for 6 cycles, and finally attained a second CR confirmed by marrow biopsy as well as PET/CT scan; Case 9 patient then received modified SMILE regimen as salvage therapy. As of the data cutoff, she was still in treatment.

References

1. Cheson BD, Ansell S, Schwartz L, et al. Refinement of the Lugano Classification lymphoma response criteria in the era of immunomodulatory therapy. Blood. 2016;128(21):2489-2496.

Figure. S1.

**Figure. S1. Best response to immunochemotherapy**

Displayed is the change in target lesion diameter from baseline (%) by magnetic resonance imaging or PET/CT. Black, progression of disease (PD); orange, stable disease (SD); blue, partial response (PR); green, complete response (CR).

Figure. S2.


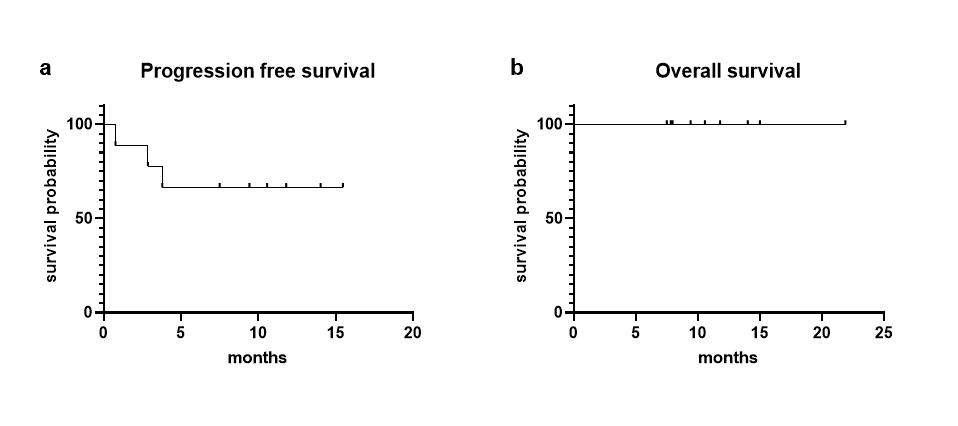


**Figure. S2. Kaplan-Meier curves of progression-free survival and overall survival of nine patients (n=9)**

Figure. S3.

**Figure. S3 Number and types of nonsilent somatic mutations (upper panel), as well as percentage of nonsilent somatic SNVs (lower panel) identified in patients with NKTCL (n = 7).**

Figure. S4.

**Figure. S4. GO-BP functional enrichment analyses of genes between the indicated two groups**

Figure. S5.


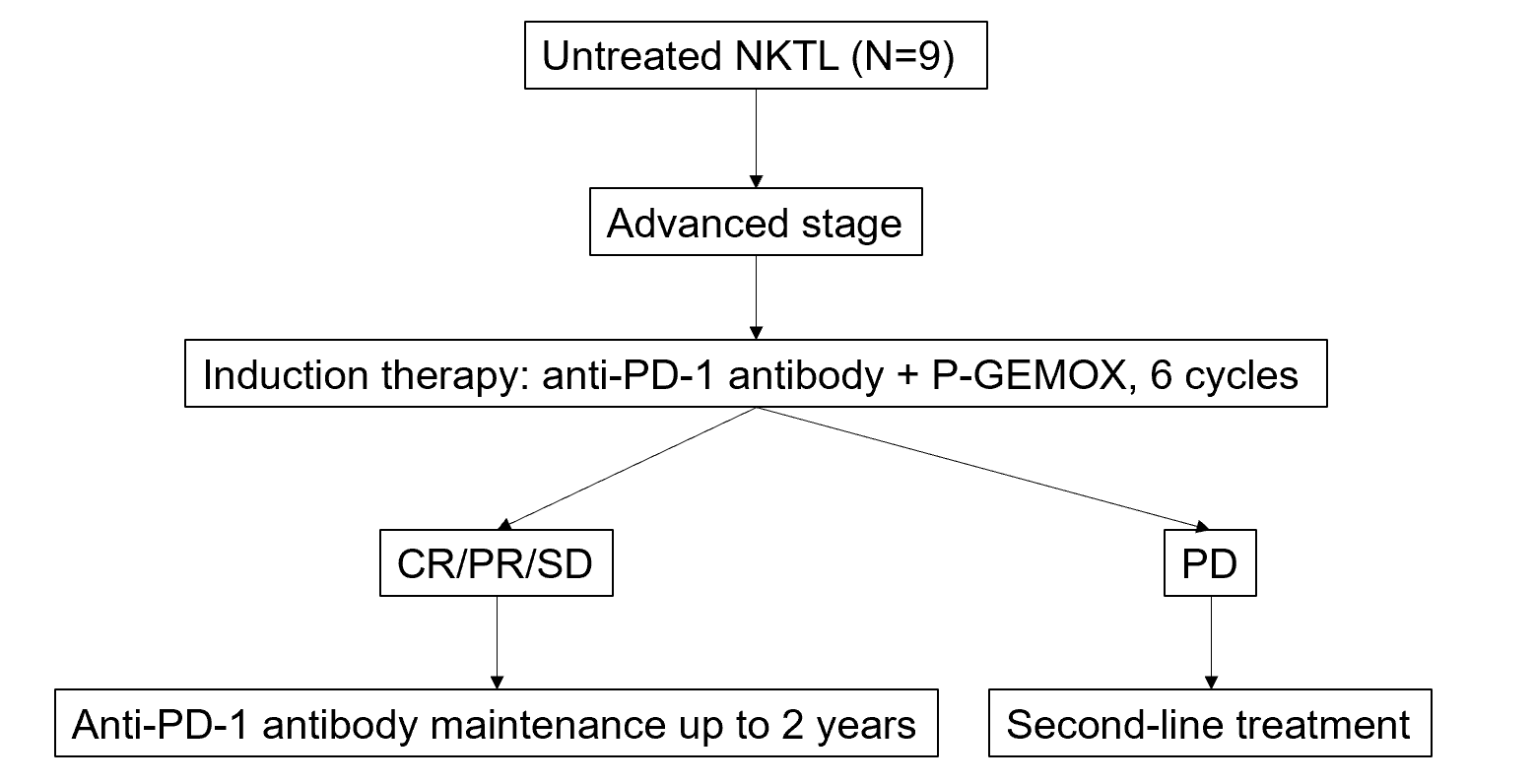


**Figure. S5. The study flowchart**

P-GemOx, pegaspargase, gemcitabine and oxaliplatin; CR, complete remission; PR, partial remission; PD, progression of disease.

Table. S1.

| Events | | n/9 (%) | | |
| --- | --- | --- | --- | --- |
| Grade 1-2 | |  | | |
| Emesis | | 4/9 (44.0%) |  |  |
| Elevated transaminase | | 4/9 (44.0%) |  |  |
| Nausea | | 3/9 (33.0%) |  |  |
| Prolonged APTT | | 3/9 (33.0%) |  |  |
| Anemia | | 2/9 (22.0%) |  |  |
| Neutropenia | | 2/9 (22.0%) |  |  |
| Diarrhea | | 1/9 (11.0%) |  |  |
| Hypothyroidism | | 1/9 (11.0%) |  |  |
| Grade 3 | |  | | |
| Anemia | | 3/9 (33.0%) |  |  |
| Neutropenia | | 3/9 (33.0%) |  |  |
| Thrombocytopenia | | 1/9 (11.0%) |  |  |
| Elevated transaminase | | 1/9 (11.0%) |  |  |
| Grade 4 | |  | | |
| Thrombocytopenia | | 2/9 (22.0%) | | |

**Table. S1. Adverse events related to the immune-chemotherapy (n=9)**

Table. S2.

| **Case** | **CD3** | **CD4** | **CD8** | **CD56** | **EBER** |
| --- | --- | --- | --- | --- | --- |
| 2 | + | - | - | + | + |
| 3 | + | + | + | + | + |
| 4 | - | - | + | + | + |
| 5 | + | - | + | + | + |
| 6 | + | - | + | - | + |
| 8 | + | - | +/- | + | + |
| 9 | + | - | - | + | + |
| +/-: weakly positive; EBER: Epstein-Barr virus-encoded RNA | | | | | |

**Table. S2. Pathological characteristics of the 7 patients with available tissue samples**
